# Development of Composite Ceramic Membranes for Carbon Dioxide Detection

**DOI:** 10.3390/membranes15100315

**Published:** 2025-10-15

**Authors:** Midilane Sena Medina, Eliana Navarro dos Santos Muccillo, Reginaldo Muccillo

**Affiliations:** Center of Science and Technology of Materials, Energy and Nuclear Research Institute, Cidade Universitária, Av. Prof. Lineu Prestes, 2242, São Paulo 05508-000, SP, Brazil; midilanelsena@gmail.com (M.S.M.); enavarro@usp.br (E.N.d.S.M.)

**Keywords:** carbon dioxide, electrochemical sensor, ceramic membranes, lanthanum molybdenum oxide, lithium sodium potassium carbonate

## Abstract

Porous La_2_MoWO_9_ (W-LAMOX) impregnated with a eutectic mixture of lithium, sodium, and potassium carbonate (LNKC) ceramic membranes was synthesized and evaluated for carbon dioxide (CO_2_) sensing applications. Structural, microstructural, and electrical characterizations were carried out using X-ray diffraction (XRD), scanning electron microscopy (SEM), and impedance spectroscopy. The results indicate that sintered thinner membranes, prepared by the tape casting method, exhibit faster and more reproducible responses to CO_2_ exposure than sintered thick pressed pellets. These findings highlight the potential of these composite membranes for application in CO_2_ sensing technologies.

## 1. Introduction

The intensification of carbon dioxide (CO_2_) emissions over recent decades has been widely associated with the enhancement in the greenhouse effect and global climate change, establishing it as one of the most critical environmental challenges of our time. Continuous and accurate monitoring of CO_2_ concentrations across various environments has therefore become a strategic necessity, both on a global scale and for industrial and laboratory applications. Traditionally, techniques such as infrared spectroscopy and gas chromatography have been employed for this purpose, offering high sensitivity and reliability. However, these methods require expensive instrumentation, complex operation, and dedicated laboratory infrastructure, in addition to presenting limitations for real-time measurements and operation under extreme temperature conditions. Carbon dioxide sensors have been developed recently [1,2,3,4,5,6,7,8,9]. A recent communication related the CO_2_ content in hospital clinical rooms to the presence of COVID-19 infection; it has been shown that an increase in the atmospheric carbon dioxide concentration (e.g., 800 ppm) is related to SARS-CoV-2 aerostability, namely, how stable and infectious that virus remains in the ambient air [10]. This was an additional reason for the research work here described.

In this context, electrochemical sensors have emerged as a promising alternative, as they enable fast, selective, and low-cost measurements, with potential for operation in harsh environments [11,12,13,14,15,16,17]. Potentiometric devices incorporating solid electrolytes and auxiliary carbonate phases have attracted attention due to their simple construction and good reproducibility. The introduction of ceramic electrolytes, such as tungsten-doped lanthanum molybdates (W-LAMOX), in combination with eutectic mixtures of lithium, sodium, and potassium carbonates (LNKC), represents an innovative approach to enhancing ionic conductivity and electrochemical stability. W-LAMOX was chosen due to its oxide ion conductivity being higher than that of the paradigmatic ZrO_2_:8 mol%Y_2_O_3_ (8YSZ) solid electrolyte [18]. LNKC was chosen due to its low melting point in comparison with other eutectic alkali halide carbonate compositions [19].

The electrochemical CO_2_ detection principle is based on the ${CO}_{3}^{2-}$ ion conduction in the molten impregnated eutectic carbonate composition (e.g., LNKC at 397 °C), through the pores of an oxide-ion-conducting solid electrolyte (e.g., W-LAMOX) [18].

Accordingly, this work aimed to investigate the preparation and the structural and electrical characterization, as well as the sensing response, of composite ceramic membranes obtained via different processing routes (bulk and tape casting), impregnated with LNKC. The majority of the research work on composite dual ceramic membranes focuses on the capture of CO_2_, not taking advantage of their application as ambient sensors. This study sought to correlate the physicochemical and microstructural properties of the materials with their performance in CO_2_ detection, with the goal of contributing to the development of sensitive and reproducible devices capable of meeting the growing demand for efficient monitoring.

## 2. Materials and Methods

### 2.1. Preparation of W-LAMOX

The compound La_2_MoWO_9_ (W-LAMOX) was synthesized via the polymeric precursor method [11]: stoichiometric amounts of molybdenum trioxide (MoO_3_–99%–Sigma Aldrich, St. Louis, MO, USA) and tungsten trioxide (WO_3_–99.8%–Alfa Aesar, Haverhill, MA, USA) were dissolved in a 5% ammonium hydroxide solution (NH_4_OH–28 vol.%–CRQ, Caravelas, Brazil) at room temperature and at 200 °C. Lanthanum nitrate hexahydrate (La(NO_3_)_3_·6H_2_O–99.999%–Sigma Aldrich) was dissolved in distilled water at room temperature. Subsequently, these solutions were added to a citric acid solution (C_6_H_8_O_7_–99.5%–Synth, São Paulo, Brazil), maintaining a molar ratio of 1:2 between citric acid and total metal cations. After stirring the mixture for 30 min at 100 °C, ethylene glycol (1,2-diol, C_2_H_4_(OH)_2_–Vetec, São Paulo, Brazil) was added, maintaining a citric acid to ethylene glycol mass ratio of 60:40. The pH of the final solution was adjusted to 2 using nitric acid (HNO_3_–Synth, Brazil), and the solution was kept under constant stirring at 100 °C for several hours until gelation occurred. The resulting gel was calcined at 200 °C for 2 h, followed by calcination at 550 °C for 4 h, using a heating/cooling rate of 5 °C/min.

### 2.2. Preparation of Porous Matrices

Porous W-LAMOX solid electrolyte ceramic matrices were prepared either by pressing (bulk sample) or by the tape casting technique (tape-cast sample). Polymethyl methacrylate (PMMA–Tokyo Chemical Industry, Tokyo, Japan) was used as a sacrificial phase before pressing. After pressing, that sacrificial phase was thermally removed to produce a pore network to be filled up with the molten carbonate. Mixtures of W-LAMOX and PMMA in volume ratios of 70/30, 50/50 and 30/70 were dispersed in an aqueous medium under mechanical stirring with zirconia milling media for 24 h. After drying, the resulting powders were uniaxially pressed into pellets with a diameter of 14.0 mm and a thickness of 3.0 mm, under a pressure of 70 MPa. The pellets were then sintered under various temperature and time conditions. The apparent density of the sintered bodies was determined by the Archimedes method using a Mettler Toledo AG245 balance (Mettler Toledo, Columbus, OH, USA). The W-LAMOX ceramic electrolyte obtained via the tape casting technique was prepared using two distinct methods for porosity induction: by incorporating in a slurry organic additives or rice starch, both as pore-forming agents. The slurry formulation was carried out as follows: initially, the W-LAMOX ceramic powder, synthesized via the polymeric precursor method, was dispersed in a mixture of ethanol (EtOH, 10.9 wt.%, Synth, Brazil), methyl ethyl ketone (MEK, 21.0 wt.%, Sigma Aldrich, USA), and polyvinylpyrrolidone (PVP, Synth), using zirconia milling media for 48 h. Subsequently, polyvinyl butyral (PVB 4.1 wt.%, Solutia Inc., Saint Louis, MO, USA), polyethylene glycol 400 (PEG400–Merck KGaA, Darmstadt, Germany), and dibutyl phthalate (DBP 99%–Sigma Aldrich, USA) were added to the mixture. The resulting suspension was mixed in a mechanical mixer (Turbula T2C, Willy A. Bachofen A.G. Maschinenfabrik, Basel, Switzerland) for approximately 60 h. The W-LAMOX ceramic electrolyte, synthesized via the polymeric precursor method, was prepared by tape casting using two slurry formulations: with and without the addition of rice starch as pore-forming agent. In both cases, the ceramic powder content was 59.2 wt.% of the total composition. Ethanol and methyl ethyl ketone were employed as solvents to promote dissolution of the organic additives and to adjust the viscosity of the suspension. Polyvinylpyrrolidone (PVP, 0.9 wt.%, Synth, Brazil) was used as dispersant. For homogenization, the ceramic powder was initially dispersed in the solvent/PVP mixture using zirconia milling media for 48 h. Afterwards, polyvinyl butyral was incorporated as the primary binder, together with dibutyl phthalate (DBP, 3.5 wt.%, 99%, Sigma Aldrich, USA) and polyethylene glycol 400 as plasticizers to improve tape flexibility. In the formulation containing the pore former, rice starch (3.0 wt.%) was added. The resulting suspension was mixed in a mechanical mixer for approximately 60 h. After this mixing period, the suspension was left to rest for 12 h and then degassed under vacuum to remove air bubbles. Tape casting was performed on a polymeric substrate, with a casting speed of 5 rpm and a controlled thickness using a doctor blade equipment set to an opening of 1.5 mm. After drying, the green tapes were cut into disc shapes and subsequently sintered. Sintering of W-LAMOX green tapes, previously cut into disc shapes, was carried out in air using a resistive furnace (Lindberg BlueM model BF51634PC-1, Thermo Fisher Scientific, Waltham, MA, USA) by (i) heating at 1 °C/min up to 200 °C without a dwell, (ii) heating at 0.5 °C/min up to 700 °C without a dwell, (iii) heating at 1 °C/min up to 1050 °C, followed by a 2 h dwell at the final temperature. After sintering, controlled cooling was performed in air at a rate of 5 °C/min.

### 2.3. Impregnation of Porous Matrices with LNKC

Porous matrices were impregnated with a eutectic mixture of Li_2_CO_3_ (43.5 mol%, 99%, Alfa Aesar, Haverhill, MA, USA), Na_2_CO_3_ (31.5 mol%, 99%, Alfa Aesar, USA), and K_2_CO_3_ (25 mol%, Alfa Aesar, USA)-LNKC. The carbonate content was determined from sample porosity, calculated using the apparent density. The amount of LNKC required to fill the membrane pores was calculated from the apparent porosity of each sample. For impregnation, a carbonate pellet was placed on the top surface of the porous matrix and heated at 500 °C for 1 h. Impregnation was assessed by FEG-SEM and EDS analyses.

### 2.4. Characterization Techniques

Density was determined by the Archimedes method using a Mettler Toledo AG245 precision balance (Columbus, OH, USA). Differential thermal analysis (DTA) was employed to determine the burnout temperatures of PMMA and rice starch. W-LAMOX–PMMA and W-LAMOX–starch composites were analyzed by thermogravimetric analysis (TGA) and differential thermal analysis (DTA) using a simultaneous thermal analyzer (Netzsch STA 409E, Selb, Germany) and α-Al_2_O_3_ as reference. Measurements were carried out under synthetic air from room temperature to 1000 °C with heating/cooling rates of 5 °C min^−1^.

Structural characterization of synthesized W-LAMOX was performed by X-ray diffraction (XRD). XRD data were collected on a D8 Advance diffractometer (Bruker-AXS, Karlsruhe, Germany) with Cu-k_α_ radiation in the 15–80° (2θ) range, using a step size of 0.05° and 3 s per step. Lattice parameters were refined by the Le Bail method using GSAS/EXPGUI [20] and CIF No. 1,533,392 from the Crystallography Open Database.

Porous matrices were examined by field-emission scanning electron microscopy (FEG-SEM, Inspect F50, FEI, Brno, Czech Republic). Pore size distribution was determined with ImageJ Version 1.34N [21]. After impregnation, the external and fracture surfaces of the matrices were analyzed by FEG-SEM coupled with energy-dispersive X-ray spectroscopy (Octane Elect Plus EDAX system, Pleasanton, CA, USA).

Impedance spectroscopy analysis was carried out in porous and impregnated ceramic membranes from 300 to 650 °C with gold disks as electrodes, applying 200 mV in the 10 Hz–10 MHz frequency range using two impedance analyzers: Hewlett Packard 4192A (HP Inc., Palo Alto, CA, USA) and Solartron SI 1260 (Ametek Solartron Metrology, Bognor Regis, UK). The samples were spring-loaded in an alumina holder placed inside a Lindberg-Blue M tubular furnace, with 1 m platinum leads connected to the impedance analyzer. Temperature was monitored with a K-type (chromel–alumel) thermocouple with its tip positioned close to the sample.

The experimental setup for voltage–time measurements was adapted according to the sample preparation method (bulk or tape cast).

(a) Bulk samples: a sensor cell was prepared using a porous W-LAMOX ceramic disk impregnated with LNKC (10 mm diameter, 2 mm thickness). The sensor element was fixed to the end of a quartz tube with high-temperature ceramic adhesive (Ceramabond^®^ 552, Aremco Products Inc., Valley Cottage, NY, USA). Electrical contact with the exposed surface was established using a platinum mesh pressed by a spring-loaded mechanism. The assembly was placed in a split-tube furnace. The electrochemical cell was configured as follows:

CO_2_(g), Pt | W-LAMOX + (Li,Na,K)_2_CO_3_ | Pt, air (1)

Platinum electrodes served as electrochemical interfaces, with the CO_2_-exposed side as the working electrode and the air side as the reference electrode. Measurements were performed at 480 °C, monitored with a K-type thermocouple positioned close to the sample. Gas flow was controlled by an MKS mass-flow controller [MKS Inc. Co., Andover, MA, USA]. Voltage and temperature data were collected with an Agilent 34972A multichannel system (Agilent Technologies, Santa Clara, CA, USA) recorded in LabVIEW (National Instruments, Austin, TX, USA).

(b) Tape-cast samples: porous W-LAMOX matrices, prepared using either corn or rice starch, impregnated with LNKC (14 mm diameter, 1 mm thickness) were fixed to a ceramic tube with high-temperature adhesive. The tube was mounted in a pressurized chamber (3 atm) equipped with independent gas inlets, platinum electrodes, and a thermocouple. The chamber was placed inside a split-tube furnace. The cell configuration was as follows:

CO_2_, N_2_(g), Pt | W-LAMOX + (Li,Na,K)_2_CO_3_ | Pt, Ar(g) (2)

After heating to 520 °C, CO_2_ was introduced into the working electrode side at a flow rate of 100 mL·min^−1^ of pure gas, while argon flowed on the reference side. The O_2_ partial pressure and humidity were not actively controlled; therefore, the measurements were carried out under nominally dry, CO_2_-rich conditions, with only residual O_2_ and H_2_O traces from the feed lines and ambient atmosphere.

A schematic diagram of the experimental setup for gas permeation analysis was published elsewhere [22].

## 3. Results

### 3.1. W-LAMOX Bulk Samples: Structural, Microstructural, and Electrical Analysis

**Figure 1:**
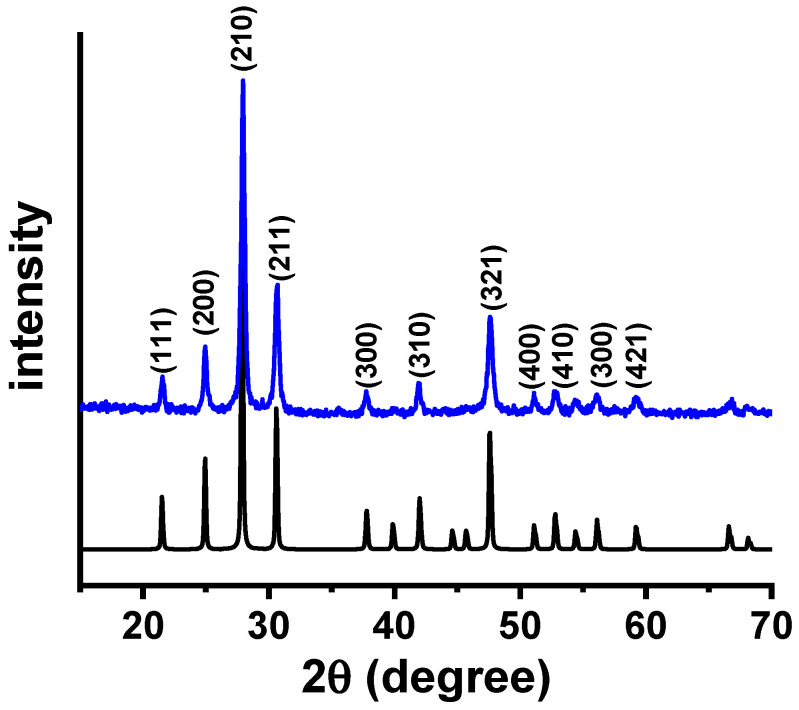
X-ray diffraction patterns of W-LAMOX synthesized by the polymeric precursor method and calcined at 550 °C/4 h (**blue**) and ICDD file 000-23-1145 (**black**).

Figure 1 shows the X-ray diffraction pattern of W-LAMOX synthesized by the polymeric precursor method after calcination at 550 °C for 4 h. Since LAMOX undergoes an α–β phase transition at 580 °C, characterized by the splitting of the (321) reflection at 2θ = 47.632° [23], the absence of such splitting in the W-LAMOX diffractogram indicates stabilization of the cubic β-phase at room temperature. The Bragg reflections observed in Figure 1 are consistent with ICDD file 000-23-1145 (space group P2_1_3), and the lattice parameter refined by the Le Bail method was a = 7.421 Å, in agreement with reported values for cubic LAMOX [24].

**Figure 2:**
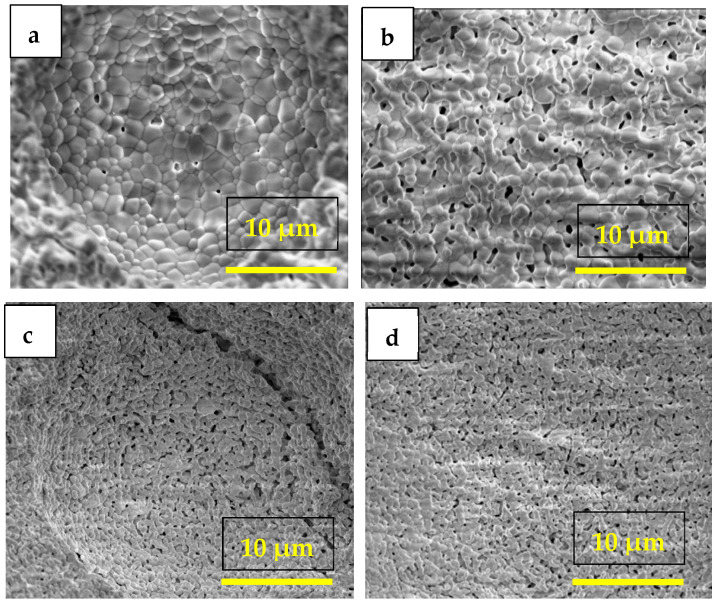
Scanning electron microscopy micrographs of porous W-LAMOX prepared with different PMMA content: (**a**) 30 vol.%, (**b**) 50 vol.%, (**c**) 70 vol.% after conventional heat treatment and (**d**) 70 vol.% after heat treatment with a reduced heating rate.

The microstructure of porous matrices prepared with different W-LAMOX/PMMA volume ratios was examined by scanning electron microscopy. Representative images after PMMA burnout are shown in Figure 2. These analyses allowed for optimization of the PMMA content and sintering conditions for obtaining densified W-LAMOX matrices.

Samples prepared with 30, 50, and 70 vol.% PMMA were initially heat-treated in two steps: 425 °C/2 h for PMMA removal and 1000 °C/2 h for sintering. The 30 vol.% PMMA samples exhibited negligible porosity (Figure 2a), whereas the 50 and 70 vol.% PMMA samples displayed adequate porosity (Figure 2b,c), more pronounced in the latter. However, both showed extensive cracking, attributed to the heating rate during PMMA burnout. To mitigate this issue, a modified thermal treatment was applied: 325 °C/2 h at 0.5 °C min^−1^, followed by 425 °C/2 h at 1 °C min^−1^ for PMMA removal, and sintering at 1000 °C/2 h. Under these conditions, the 70 vol.% PMMA sample exhibited appropriate porosity and densification without crack formation (Figure 2d).

**Figure 3:**
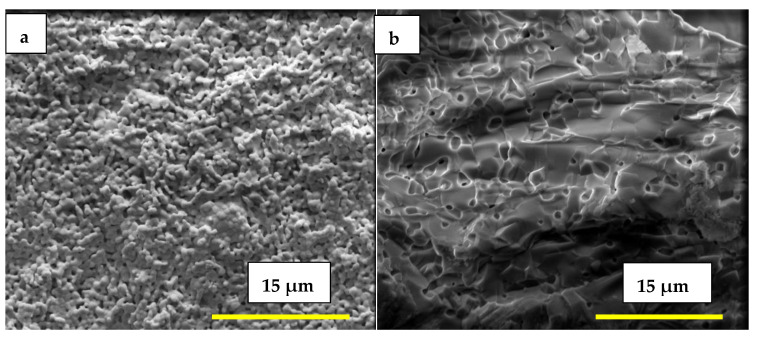
Scanning electron microscopy micrographs of porous W-LAMOX after LNKC impregnation: (**a**) external surface (**b**) fracture surface; bar length 15 µM.

The FEG-SEM micrographs in Figure 3a,b, taken, respectively, from the external surface and fracture surface of the porous matrix impregnated with the eutectic (Li,Na,K)_2_CO_3_ mixture at 500 °C, show a dense solid electrolyte matrix partially coated with carbonates and with the pore network fully filled, as observed via EDS analysis.

**Figure 4:**
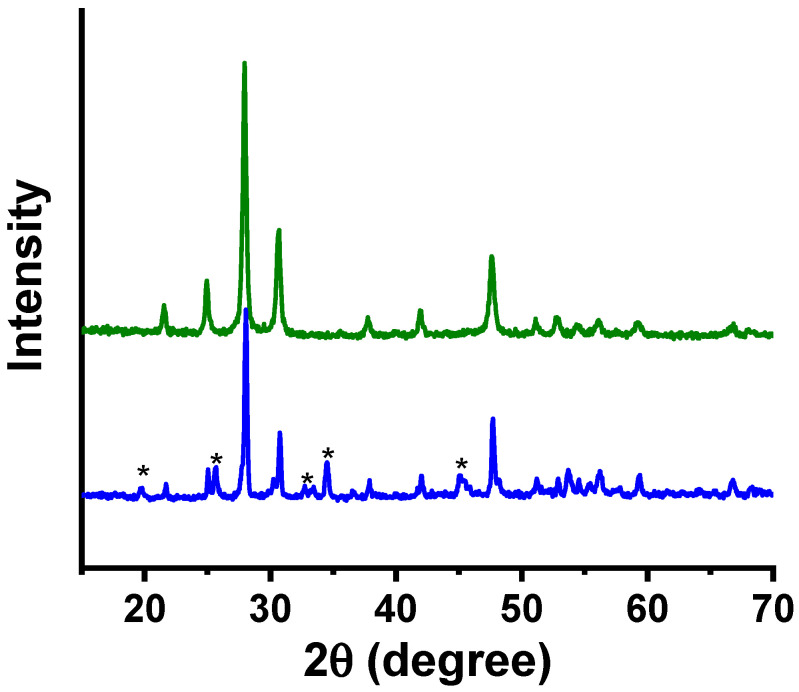
X-ray diffraction patterns of porous W-LAMOX (**olive**) and W-LAMOX impregnated with LNKC (**blue**); LNKC reflections (*).

To evaluate the structural stability of W-LAMOX after impregnation with LNKC, XRD analysis was performed on W-LAMOX/LNKC. The diffractogram (Figure 4) shows that the positions and intensities of the W-LAMOX Bragg reflections remain essentially unchanged compared with pristine W-LAMOX, indicating the absence of reactions between the ceramic matrix and the molten carbonates during impregnation. The additional peaks, marked with asterisks, correspond to the LNKC phase.

**Figure 5:**
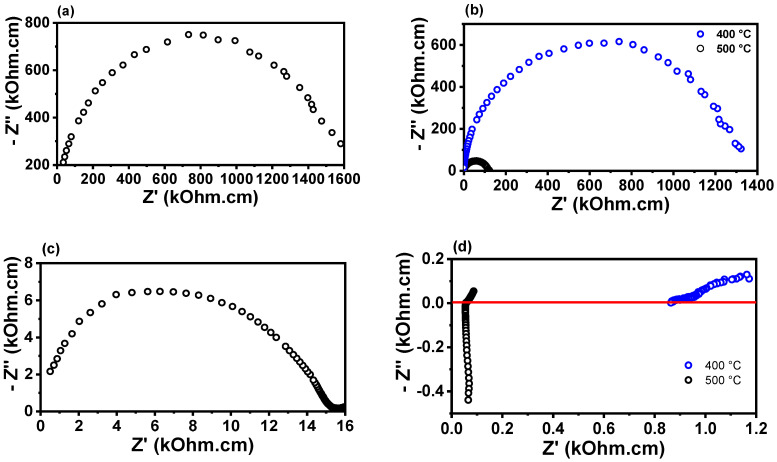
Impedance spectroscopy diagrams of porous W-LAMOX (**a**,**c**) and W-LAMOX/LNKC (**b**,**d**) impregnated at 400 °C/1 h and 500 °C/1 h; temperature of measurement: 350 °C (**a**,**b**) and 500 °C (**c**,**d**).

Figure 5 shows the impedance diagrams for porous W-LAMOX (a,c) and W-LAMOX/LNKC (b,d), impregnated at 400 °C (blue curve) and 500 °C (black curve), measured at approximately 350 °C and 500 °C. At ~350 °C, the impedance diagrams exhibit an arc-shaped profile, with no clear distinction between grain and grain boundary contributions. It is observed that the total (grain + grain boundary) resistivity of W-LAMOX/LNKC impregnated at 500 °C is approximately one order of magnitude lower than the total resistivity of porous W-LAMOX and W-LAMOX/LNKC impregnated at 400 °C. These results indicate that, for samples impregnated at 500 °C, pore filling promotes an increase in total conductivity, attributed to the reduction in microstructural defects and the synergistic effect of the two phases in ionic conduction. At 500 °C, the diagram of porous W-LAMOX retains its arc shape, whereas the W-LAMOX/LNKC diagram shows a significant change, indicating an increase in conductivity above the melting temperature of LNKC. This behavior confirms that, above this temperature, conduction is dominated by the molten LNKC infiltrated into the pores.

**Figure 6:**
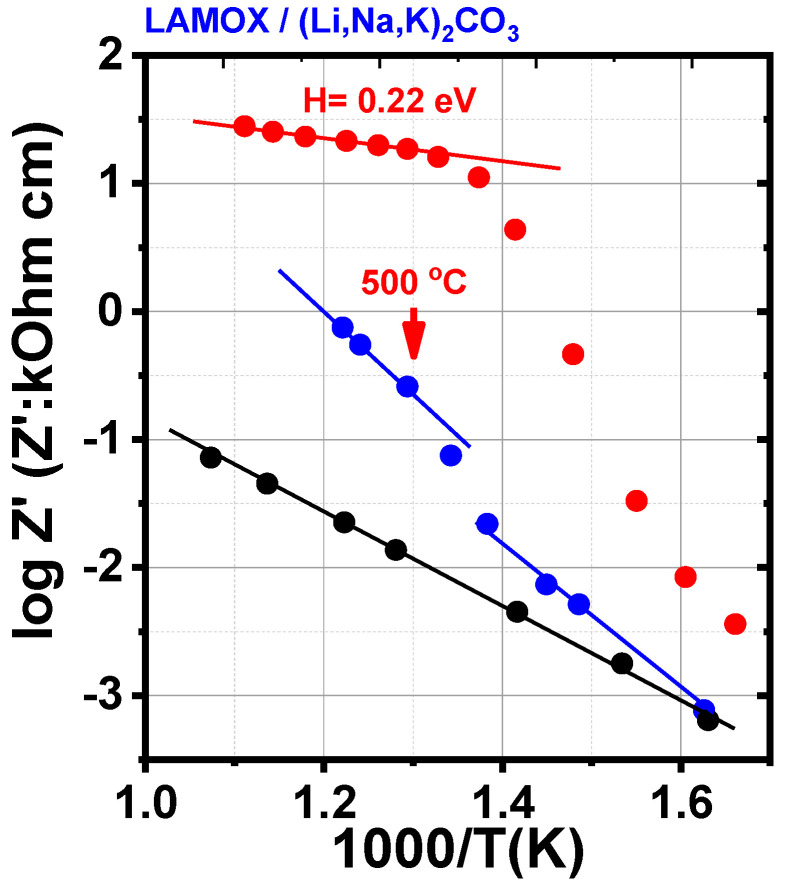
Arrhenius plots of the electrical resistivity of W-LAMOX porous membrane (**black**), impregnated with LNKC at 400 °C (**blue**) and impregnated with LNKC at 500 °C (**red**).

The porous matrix exhibits Arrhenius behavior across the entire analyzed temperature range (Figure 6), with an activation energy (H) of 1.3 eV, which is close to previously reported values for W-doped LAMOX [18]. For the porous matrices impregnated with LNKC, the conductivity curve as a function of the reciprocal of temperature reveals three distinct regions: below ~420 °C (sample impregnated at 400 °C) and ~370 °C (sample impregnated at 500 °C), a typical Arrhenius behavior is observed; above these temperatures, there is a sharp increase in conductivity, accompanied by a discontinuity in the Arrhenius regime. This phenomenon is associated with the partial melting of LNKC, which promotes contact between the two phases, enhancing ionic transport and resulting in the observed increase in conductivity [25,26]; at higher temperatures, above ~430 °C, linearity is reestablished, and conductivity once again follows Arrhenius behavior. In this region, the activation energies obtained were 1.0 eV and 0.22 eV for W-LAMOX/LNKC impregnated at 400 °C and 500 °C, respectively. The activation energy for W-LAMOX/LNKC impregnated at 500 °C is lower than that reported for the eutectic mixture of LNKC with the same mass ratio used in this study [18], probably due to loss of molten LNKC from the porous matrix. Impregnation at 400 °C is more effective because it was performed at the melting point of LNKC.

These results show that, regardless of the impregnation temperature, the combination of W-LAMOX with molten LNKC contributes to an increase in conductivity across the entire evaluated temperature range. However, impregnation at 500 °C proved to be more effective in promoting the infiltration of molten carbonates into the porous matrix. As observed in other solid oxides used as composite membranes, the evaluated activation energy confirms that, above the melting temperature of LNKC, ionic conduction is predominantly governed by the molten carbonates [18].

### 3.2. W-LAMOX by Tape Casting: Structural, Microstructural, and Electrical Analysis

**Figure 7:**
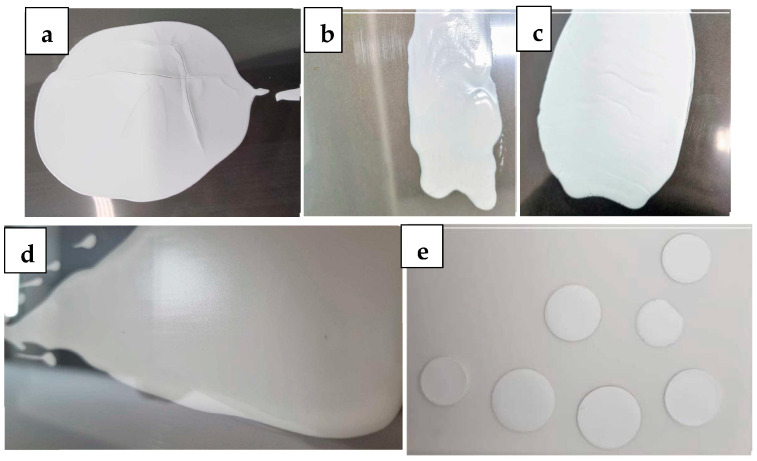
Images of surfaces of W-LAMOX tape-cast pieces: (**a**) without pore forming agents, (**b**,**c**) with corn starch pore former, (**d**) with rice starch pore former, and (**e**) dye cut pieces from (**c**).

Tape-cast samples, prepared with and without a pore-forming agent (corn or rice starch), were analyzed. Figure 7a shows pictures of W-LAMOX prepared without a pore former, where cracking defects can be observed. The intact regions of the tape were cut into disks, heat-treated, and their densities were evaluated. These images indicated that the samples exhibited low porosity (~25%). To increase porosity, different amounts of corn starch were introduced into the ceramic slurry, without success, with tape casting resulting in pieces with irregular texture (Figure 7b,c). Further tests were conducted by replacing corn starch with rice starch. This substitution enabled the production of homogeneous tapes without irregularities, with a porosity level, estimated from density measurements (~60%), suitable for impregnation.

**Figure 8:**
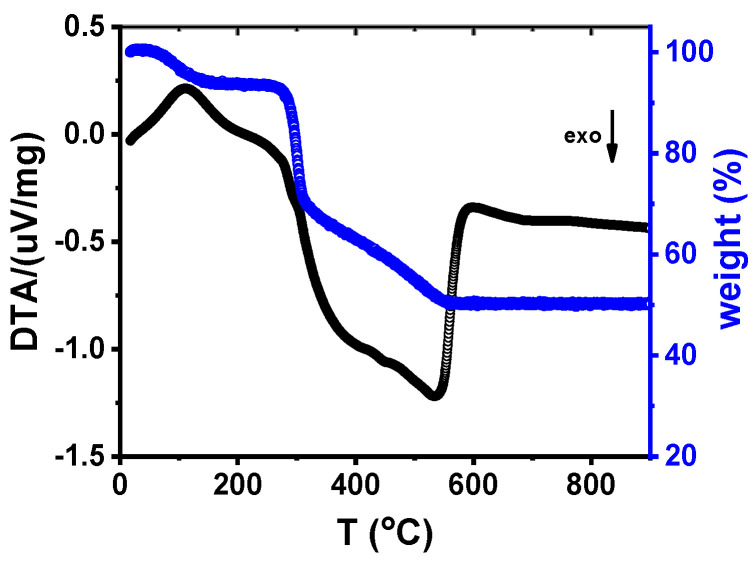
Thermogravimetric and differential thermal analysis curves of W-LAMOX mixed with rice starch.

To determine the temperature required for rice starch removal, a W-LAMOX/rice starch mixture was subjected to TG/DTA analysis. Figure 8 shows the results of the mixture obtained by differential thermal analysis (DTA) and thermogravimetric analysis (TG). An initial small mass loss is observed up to approximately 150 °C, attributed to the elimination of adsorbed moisture on the powder surface. From 300 °C onwards, a pronounced mass loss occurs, particularly in the 300–550 °C range, associated with the degradation of rice starch, the organic pore-forming agent. This decomposition is accompanied by a distinct exothermic peak in the DTA curve, indicating heat release during the combustion of the organic material.

In the 400–600 °C range, a continued but less pronounced mass loss is observed, likely associated with the oxidation of carbonaceous residues from starch decomposition. Above 700 °C, the TG and DTA curves stabilize, suggesting that the decomposition processes are complete. Based on these results, the heating schedule for W-LAMOX prepared by tape casting was defined in three stages: (1) 1 °C/min up to 200 °C (no dwell), (2) 0.5 °C/min up to 700 °C (no dwell), and (3) 1 °C/min up to 1050 °C, with a 2 h dwell, followed by controlled cooling at 5 °C/min.

**Figure 9:**
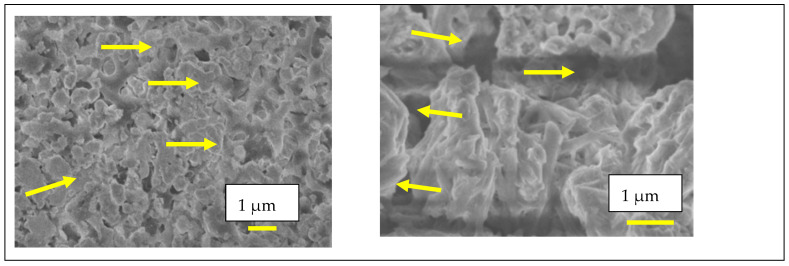
Scanning electron microscopy micrographs of tape casting samples: (**right**) porous W-LAMOX after thermal treatment for remotion of organic compounds; (**left**) porous W-LAMOX impregnated with LNKC (yellow arrows); bar size = 1 µM.

Microstructural analysis (Figure 9) reveals significant differences between the porous sample and the sample impregnated with LNKC. The image on the right shows the porous microstructure obtained by tape casting, characterized by a high degree of interconnected porosity. In contrast, the image on the left depicts W-LAMOX after impregnation with LNKC, where carbonates are observed to be distributed throughout the porous matrix, coating the structure and reducing the apparent porosity. This microstructural modification is essential to ensure adequate ionic conductivity and gas permeation through the porous matrix.

**Figure 10:**
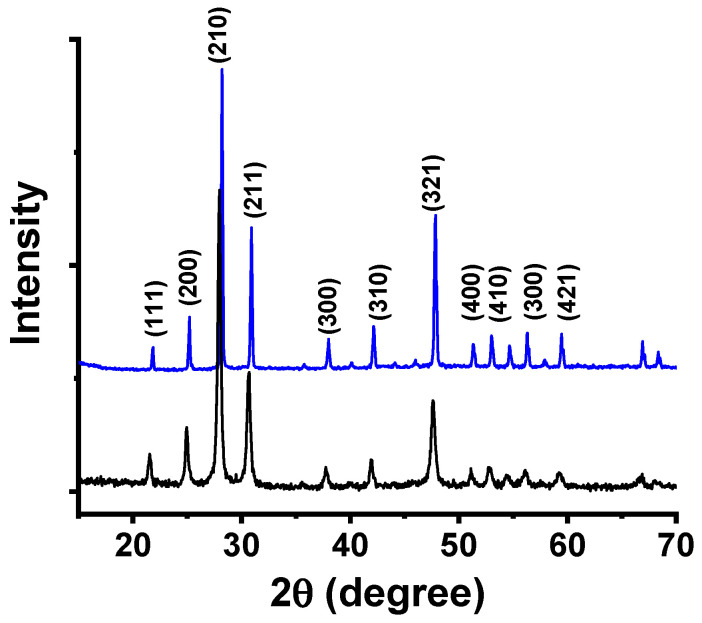
X-ray diffraction patterns of porous tape-cast (**blue**) and pressed pellet (**black**) W-LAMOX.

Figure 10 compares the X-ray diffraction pattern of W-LAMOX obtained by tape casting with that of the bulk sample. The main reflections characteristic of the cubic crystalline structure, at 2θ ~27° (210), ~32° (211), and ~46° (321), are present in both diffractograms, indicating that the tape-casting process did not alter the crystalline phase of W-LAMOX. Few small reflections, probably due to secondary phases, could not be indexed. The relative peak intensities are higher for the tape-casting sample, suggesting improved crystallinity or enhanced preferred orientation. In addition, the full width at half maximum (FWHM) of the peaks is slightly smaller, which may be associated with an increase in average crystallite size. These variations can be correlated with the microstructure resulting from the processing method.

The X-ray diffraction results confirm the formation of the expected crystalline phase without evidence of significant secondary phases.

**Figure 11:**
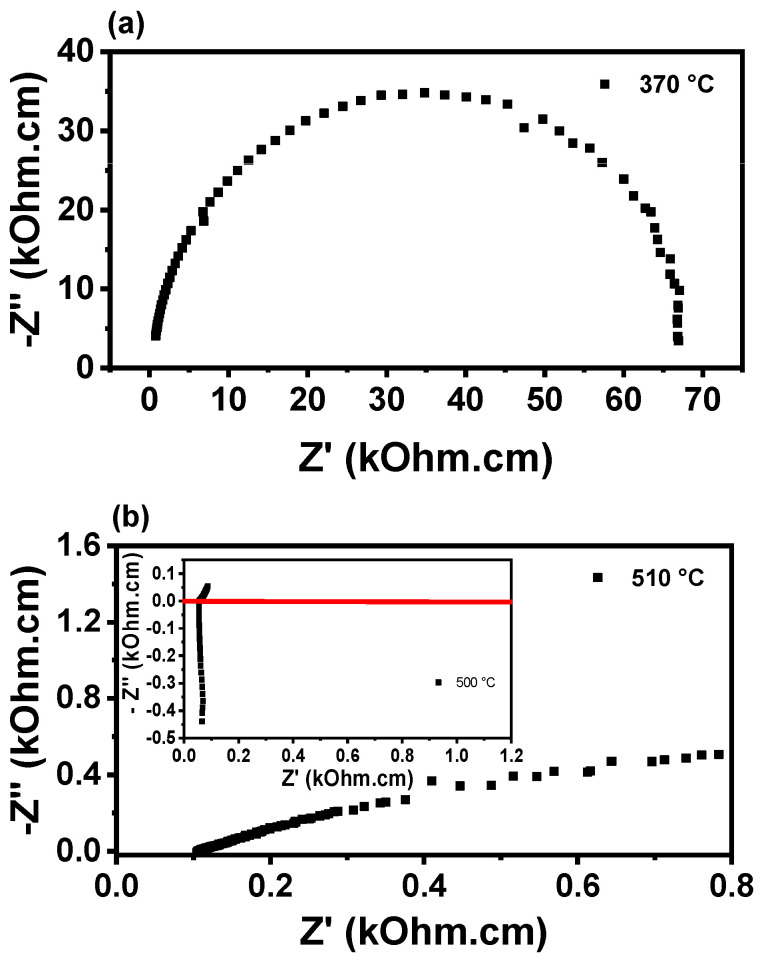
Impedance spectroscopy diagrams of W-LAMOX/LNKC impregnated with LNKC at 500 °C/1 h; measurements at (**a**) ~370 °C and (**b**) ~510 °C. Inset: impedance diagram of the bulk W-LAMOX pellet.

Figure 11 shows the impedance diagrams of the W-LAMOX/LNKC sample measured at 370 and 510 °C. A marked difference in the electrical response as a function of temperature is observed, associated with the physical state of LNKC and its interaction with the grain boundaries. At 370 °C, the spectrum displays a well-defined, large semicircular arc, indicative of high total resistance. Under these conditions, the LNKC phase is predominantly solid or only partially molten. Consequently, grain boundary wetting is limited, and ionic transport remains strongly hindered by W-LAMOX grain boundary barriers, resulting in low conductivity. At 510 °C, when the LNKC phase is molten, a pronounced decrease in impedance is observed. The liquid phase percolates through the pores and along the grain boundaries, thereby reducing grain boundary resistance, increasing the effective interfacial contact area between W-LAMOX and LNKC, and enhancing fast ionic transport. This leads to the suppression of the semicircular arc and a clear increase in conductivity.

A comparison of impedance results for tape-cast samples (~1 mm thickness) and bulk samples (~7 mm thickness) in the inset, measured at similar temperatures (~500 °C), highlights notable differences. The bulk sample exhibits significantly lower resistance (Z′ ~0.1 kΩ·cm), suggesting the formation of well-established conductive pathways, likely promoted by more efficient percolation of molten LNKC within its thicker microstructure. In contrast, the tape-cast sample shows a more evident semicircular arc and higher impedance values (~70 kΩ·cm), indicating enhanced resistance to ionic transport. As the ionic conductivity is proportional to the carbon dioxide ion’s density, charge (−2), and mobility, the reduced thickness limits the amount of LNKC located at the pores, reducing the charge carrier density, and consequently increasing the ionic resistivity. Overall, the results demonstrated that sample geometry and forming methods strongly influenced the electrical response of the composite membrane, with the bulk specimen being more favorable for ionic conduction at high temperatures.

### 3.3. Voltage–Time Measurements

Voltage–time measurements were carried out at approximately 500 °C to qualitatively evaluate the performance of different ceramic matrices impregnated with the eutectic LNKC mixture looking for membranes for CO_2_ detection. Substrates of W-LAMOX were compared in different thicknesses and prepared by distinct forming methods (bulk and tape casting).

**Figure 12:**
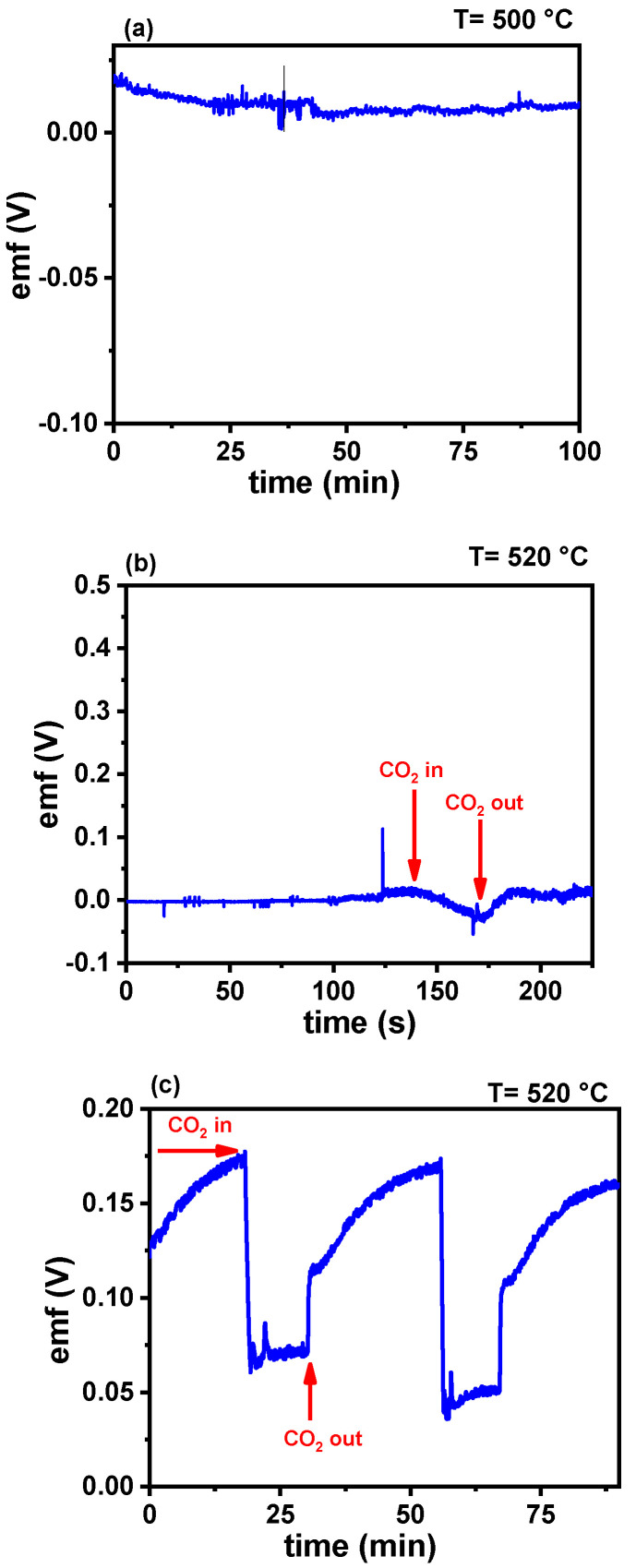
Electromotive force (emf) values evaluated with bulk W-LAMOX (**a**); bulk W-LAMOX/LNKC with (**b**) 1.7 mm and (**c**) 1.0 mm thicknesses.

In Figure 12a, the curve corresponding to porous W-LAMOX shows no electrochemical response, indicating that the ceramic matrix alone is not sensitive to CO_2_, and that the presence of molten carbonates is essential for detection. For bulk W-LAMOX/LNKC, a thickness-dependent response was observed. The 1.7 mm sample (Figure 12b) exhibited an irregular response with a longer stabilization time, whereas the 1.0 mm sample (Figure 12c) showed an improved reproducible behavior, with well-defined gas inlet/outlet cycles and amplitude in the range of 0.15–0.20 V. These results indicate that reducing the thickness favors gas diffusion between the liquid electrolyte and solid matrix, thereby improving sensor performance.

**Figure 13:**
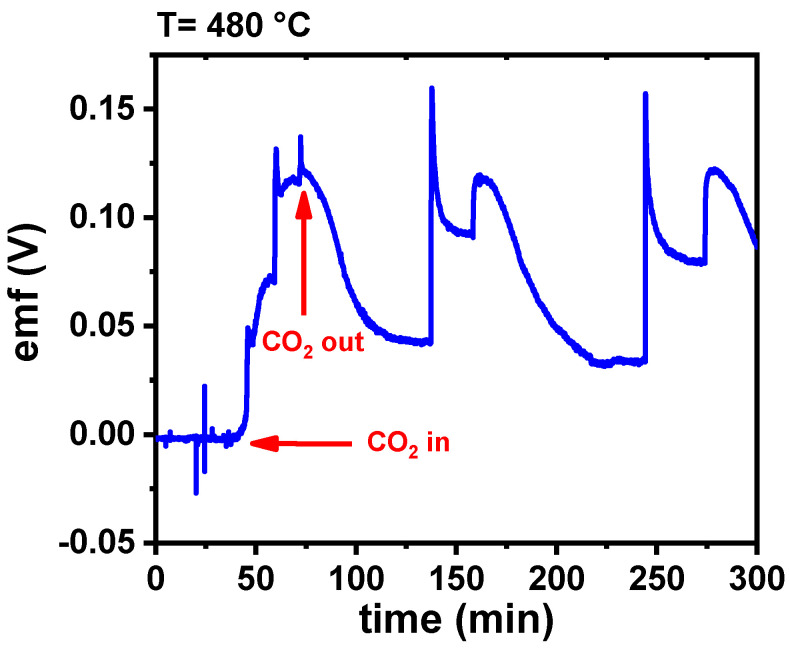
Electromotive force (emf) values of tape-cast W-LAMOX/LNKC under CO_2_ injection.

For the tape-casting samples (Figure 13), superior performance was observed, with rapid and stable responses to successive CO_2_ injection and removal cycles. In the case of W-LAMOX/LNKC, a fast recovery of the signal after gas removal was noted, which is a desirable characteristic in sensors.

The main results show that reduced sample thickness is a key factor in enhancing both sensitivity and response reproducibility. Bulk W-LAMOX/LNKC showed faster signal recovery. However, this same affinity slows down the desorption process, resulting in longer recovery time. Tape-cast W-LAMOX/LNKC, on the other hand, although it provides good ionic conductivity, contributes less electronically and exhibits lower CO_2_ adsorption at its surface. As a result, its response amplitude is smaller, but signal recovery is faster, with a prompt return to the baseline once the gas is removed.

### 3.4. Response and Recovery Times of Bulk and Tape-Cast W-LAMOX/LNKC

The response and recovery times of W-LAMOX/LNKC (bulk and tape casting) samples were evaluated using parameters *t*_90_ (time to reach 90% of the gas signal variation after CO_2_ introduction) and *t*_10_ (time to return to 90% of the baseline signal after CO_2_ removal). The results highlight the significant influence of the ceramic matrix thickness and material composition on the CO_2_ detection kinetics.

#### 3.4.1. Bulk (1.0 mm) W-LAMOX/LNKC

Exhibited slow response, with a *t*_90_ of approximately 12 min and a recovery time of ~15 min; the prolonged response is attributed to the extended diffusion path required for gas molecules to reach equilibrium within the LNKC-impregnated active region; although the signal amplitude is higher, the increased thickness limits its suitability for applications requiring rapid sensor response.

#### 3.4.2. Tape-Cast (1 mm) W-LAMOX/LNKC

Demonstrated significantly fast kinetics, with a *t*_90_ of ~3 min and a recovery time of ~5 min—approximately three times faster than the bulk sample; the reduced thickness enhances detection kinetics by facilitating quicker stabilization of the carbonate-oxide system.

#### 3.4.3. Diffusional Limitations in Bulk Samples

The long recovery time in bulk W-LAMOX is likely due to restricted gas transport within the thicker structure; greater thickness may promote CO_2_ retention in internal pores and within the molten LNKC phase, delaying signal stabilization and return to baseline potential.

The results demonstrate that reducing ceramic matrix thickness is critical for accelerating response kinetics, while the choice of ceramic material strongly affects sensitivity and recovery behavior. Among the evaluated samples, tape-cast W-LAMOX impregnated with LNKC exhibited the best performance in terms of response time, recovery, and signal stability, making it a promising candidate for CO_2_ sensor applications.

## 4. Conclusions

The development and investigation of composite W-LAMOX/LNKC ceramic membranes for carbon dioxide detection enabled the assessment of the influence of microstructure, thickness, and ceramic membrane preparation technique on sensor performance. Analyses confirmed that, above the melting point of LNKC, a significant increase in ionic conductivity occurs, evidencing the role of the molten phase in the transport of carbonate species. CO_2_ detection tests demonstrated that thinner samples exhibited faster and more reproducible responses. The results demonstrated the feasibility of using W-LAMOX/LNKC composite ceramic membranes as CO_2_ sensors. Furthermore, this study shows that tailoring microstructure through processing, combined with the appropriate choice of ceramic membrane production, constitutes a key strategy to enhance the sensitivity, selectivity, and stability of the sensor device. Thus, although it presents qualitative carbon dioxide detection, this work contributes to the advancement of efficient electrochemical sensors, aligned with the increasing demands of environmental, industrial, and medical location monitoring. Future work will explore i) the insertion of contents of CO_2_ gas at ppm level to build a calibration curve and ii) the design and building of a sensor device composed of the W-LAMOX/LNKC ceramic membrane deposited in a metallic support, with a heating element able to reach 400 °C and a LED connect to a suitable chip for indicating the CO_2_ concentration.

## Data Availability

Experimental data will be available from the corresponding author upon request.
